# Modelling the Transmission of *Coxiella burnetii* within a UK Dairy Herd: Investigating the Interconnected Relationship between the Parturition Cycle and Environment Contamination

**DOI:** 10.3390/vetsci9100522

**Published:** 2022-09-24

**Authors:** Dimitrios G. Patsatzis, Nick Wheelhouse, Efstathios-Al. Tingas

**Affiliations:** 1 Institute of Sciences and Technologies for Sustainable Energy and Mobility, National Research Council, 80125 Napoli, Italy; 2 School of Applied Sciences, Edinburgh Napier University, Edinburgh EH10 5DT, UK; 3 School of Computing, Engineering and the Built Environment, Edinburgh Napier University, Edinburgh EH10 5DT, UK

**Keywords:** Q fever, *Coxiella burnetii*, epidemiological model, dairy herd

## Abstract

**Simple Summary:**

Q fever infection in dairy herds is introduced through the transmission of the bacterium *Coxiella burnetii*, resulting in multiple detrimental effects such as reduction of lactation, abortions and chronic infection. Particularly in the UK, recent evidence suggests that the infection is endemic in dairy cattle. In this work, we investigate the dynamics of the disease with the aim to disentangle the relationship between the heterogeneity in the shedding routes and their effect on the environmental contamination. We develop a mathematical model for the transmission of Q fever within UK cattle herds by coupling the within-herd infection cycle of the disease with farm demographics and environmental effects, introduced by either the indoor or outdoor environment. The present analysis aims also to indicate the gaps in the available data required to optimise the proposed model or future models that will developed on the basis of the one proposed herein. Finally, the developed model can serve as mathematical proof for the assessment of various interventions for controlling the dynamics of Q fever infection.

**Abstract:**

Q fever infection in dairy herds is introduced through the transmission of the bacterium *Coxiella burnetii*, resulting in multiple detrimental effects such as reduction of lactation, abortions and chronic infection. Particularly in the UK, recent evidence suggests that the infection is endemic in dairy cattle. In this work, we investigate the dynamics of the disease with the aim to disentangle the relationship between the heterogeneity in the shedding routes and their effect on the environmental contamination. We develop a mathematical model for the transmission of Q fever within UK cattle herds by coupling the within-herd infection cycle of the disease with farm demographics and environmental effects, introduced by either the indoor or outdoor environment. Special focus is given on the mechanism of transmission in nulliparous heifers and multiparous cattle. We calibrate the model based on available knowledge on various epidemiological aspects of the disease and on data regarding farm demographics available in the UK DEFRA. The resulting model is able to reproduce the reported prevalence levels by *field* and *in silico* studies, as well as their evolution in time. In addition, it is built in an manner that allows the investigation of different housing techniques, farm management styles and a variety of interventions. Sensitivity analysis further reveals the parameters having the major effect in maintaining high prevalence levels of seropositive and shedding cattle. The present analysis aims also to indicate the gaps in the available data required to optimise the proposed model or future models that will developed on the basis of the one proposed herein. Finally, the developed model can serve as mathematical proof for the assessment of various interventions for controlling the dynamics of Q fever infection.

## 1. Introduction

*Q fever* or query fever is a zoonotic disease caused by the Gram-negative intracellular bacteria *Coxiella burnetii* (*C. burnetii*). The bacteria has a wide host range and can infect a number of invertebrate (e.g., ticks) and vertebrate hosts (e.g., dogs, cats, rabbits, horses, pigs, camels, rodents) [1], while there is evidence of *C. burnetii* within ticks, the prevalence particularly in Northern Europe is low [2]. There is also little evidence of transmission from wildlife to date and clinical Q fever is largely recognised as a disease of ruminant livestock across Europe, particularly within goats and sheep, which are also thought to be the main source of human infection.

Beyond the impacts of Q fever on human health, the infection has significant potential economic and welfare implications for the livestock industry. Symptoms of Q fever in ruminants may include, but are not limited to, fever, mild coughing, anorexia, rhinitis, metritis, chronic mastitis and fertility problems such as abortion, stillbirths, infertility, premature delivery and weak offspring, however, very often the infection remains apparently asymptomatic [3].

The diagnosis of clinical Q fever within cattle appears to be less common than in small ruminants, despite the apparent endemic nature in cattle across mainland Europe and the UK. However, there is evidence that the infection can cause abortion and that vaccination against the disease may improve reproductive performance of cattle, suggesting an important but subclinical role in fertility [4]. Poor fertility is one of the main reasons for culling of dairy cattle, and this combined with the potential zoonotic nature of the infection suggests that Q fever in cattle remains a largely overlooked but important disease for European agriculture and that through understanding the roles of the different potential pathways of transmission is a vital step in any future One Health control strategy.

Infection with *C. burnetii* is thought to be only through inhalation of infective material via the oronasal route, while many infected animals will remain apparently asymptomatic they may throughout their lives shed various quantities of the bacterium through a number of routes including faeces, urine, milk, vaginal mucus and most notably parturition products [5]. However, while the bacteria may be shed intermittently through all routes, it is thought that it is the process of parturition and presence of significant placental contamination that is one of the definitive risk factors for the transmission of Q fever compared to other shedding routes [5,6]. With *C. burnetii* genome copy number in placenta, $1.5\times 10^{8}$ and $2.5\times 10^{8}$ Genome Equivalents (GE)/gram, which are significantly greater than the respective values in vaginal swabs, $6.3\times 10^{5}$ GE/gram, or bulk sample from livestock living areas $2.48\times 10^{3}$ GE/gram [6].

Environmental factors such as the proportion of bacteria that shed through mucus/ faeces in the environment and the decay rate of *C. burnetii* play a significant role in infection dynamics [7]. Contamination of the surrounding environment allows the bacterium to aerosolise, infecting susceptible animals via an oronasal route. *Coxiella burnetii*’s cellular structure also permits it to survive adverse environmental conditions. The non-replicative small cell variant (SCV), can withstand both desiccation and temperature changes and is naturally resistant to a number of disinfectants [8]. These cellular properties not only allows the bacteria to persist for a significant period of time within the environment, it additionally allows the bacterium to spread in vast distances [9]. In the United Kingdom (1989), cases of Q fever have been recorded up to 11 miles (18 km) from the epicentre of the disease outbreak [10]. These intrinsic cellular properties enhance the severity of *C. burnetii*, the natural resistance to environmental decay permits the disease to both establish itself within an individual herd but also spread to other farms.

Despite the potential for extensive environmental dispersal over significant differences, it is clear that the force of infection is much stronger in certain micro-environments; e.g., birthing pens and living areas become more contaminated than pastures [6,11]. For example, Kersh et al. detected *C. burnetii* DNA levels of between 900 and 116,000 GE/gram contamination levels directly within birthing pens and 10 GE/gram within pasture at a distance of up to 30 m from birthing pens on goat farms after a Q fever outbreak [6]. Therefore, in order to fully understand the underlying infection dynamics of within-herd transmission, it is required to disentangle the interconnected relationship of the infection cycle with parturition and environmental contamination.

The available literature on the mathematical modelling of the transmission of *C. burnetii* in ruminants is particularly scarce. The stochastic compartmental model developed by Courcoul et al. [7,12] for French cattle herds considered aspects of herd management and demographics. Special emphasis was placed on the different shedding routes, hence the particular detail on the infected shedding and non-shedding classes (five sub-populations in total). In addition, the coupling effect of the environment was considered by introducing an environment compartment incorporating the added bacterial load from the shedders prevalence in each shedding route category (weighted by their respective shedding levels). The proposed model reproduced quite well the available field data and set the basis for various extensions that focused on modelling the inter-herd transmission of *C. burnetii* by wind and trade of cows [13], the investigation of abortion storms in dairy goat and cattle herds [14], as well as the effectiveness of different vaccination strategies [15]. Bontje et al. [16], rather than considering multiple infected classes, accounted for separate compartmentalisation for the infected pregnant and non-pregnant goats. More important, Bontje et al. [16] took into account the bacterial load increase in the environment due to parturition. The model of Bontje et al. [16] was not validated with experimental data, yet, it shed more light on the deciphering of the dynamics of *C. burnetii* transmission in dairy goat herds. Asamoah et al. [17] developed a model which considered three sub-populations (susceptible, asymptomatic and infected) and two other compartments; one for the environment and another for the vaccinated population. Due to its simplicity it was used for an intensive mathematical investigation, i.e., determination of the equilibrium points, local and global stability analysis, sensitivity analysis, and optimal control analysis to determine the conditions for the most effective control measures.

In light of the existing literature, it becomes evident that none of the proposed models had diversified the effects of parturition and micro-environment contamination on the within-(dairy) herd disease dynamics. The model proposed herein addresses these two crucial factors while also taking into account the effect of the farm demographics. We advocate that the consideration of these three factors (parturition, micro-environment, farm demographics) is a necessary, from a modelling perspective, condition for the production of a biologically sound outcome.

The mathematical model presented herein is meant to be a meaningful step towards improving our understanding of *C. burnetii* transmission dynamics by building upon previous literature and constructing a biologically sound deterministic model which explicitly incorporates different environmental factors and farm demography. Unlike previous modelling efforts, the proposed model explicitly incorporates different micro-environments and the typical parturition cycle for intensive dairy cattle. By utilising parameter values presented in the literature and some associated assumptions, we investigate the impact of different farm management techniques or scenarios on the within-herd disease dynamics.

## 2. Material and Methods

### 2.1. The *C. burnetii* within-Herd Transmission Model

**Figure 1:**
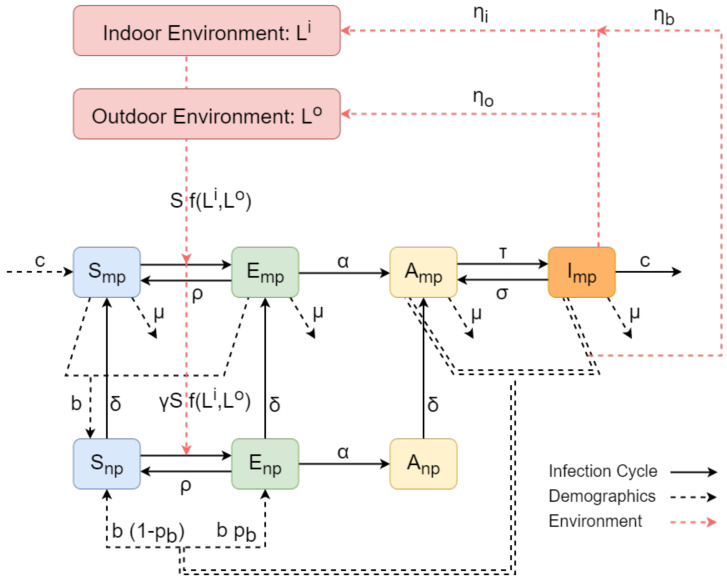
Flow diagram representation of the *C. burnetii* transmission model. The sub-populations of the model within the nulliparous and multiparous compartments are denoted by rounded squares, except from the magenta squares that denote the indoors and outdoors environment compartments. The transition rates between the sub-populations are indicated by the black arrows (solid/dashed when related to the infection cycle/demographics) with their associated parameters. The dashed red arrows denote transition rates incorporating contributions from/to the environment.

The basis of the epidemic model includes four population types, Susceptible-Exposed-Asymptomatic-Infected (SEAI), each incorporating different aspects of the bacteria spread. A recovered population has not been considered because there is no evidence supporting that an infected animal ever clears the infection but rather becomes an intermittent shedder [18]. Since parturition is one of the definitive risk factors for the transmission of *C. burnetii*, compared to other shedding routes such as milk and faeces/mucus [5,6], the first three populations (Susceptible-Exposed-Asymptomatic) are comprised of sub-populations of heifers and cows, namely the nulliparous and multiparous compartments, respectively. The dynamics of the infection cycle acts within the nulliparous and multiparous compartments, which are interconnected via farm demographics related transitions. The model also includes two additional compartments representing the indoor and the outdoor environment and consist of landscapes with different levels of contamination. The environment compartments interconnect with the nulliparous and multiparous ones for enabling *C. burnetii* transmission through the different shedding pathways. The outline of the Q fever transmission model is presented in the flow diagram representation of Figure 1, where the sub-populations within the nulliparous and multiparous compartments are displayed in detail.

#### 2.1.1. The Infection Cycle

The infection lifecycle of Q fever within the nulliparous and multiparous compartments is modelled by cattle sub-populations of different health status. The susceptible cattle, *S*, have never been in contact with the bacterium, while the cattle that have faced an initial exposure are considered exposed, *E*. The infected cattle, which are not yet shedding or have any other symptom are considered asymptomatic *A*, while the ones that have begun shedding are considered infectious, *I*. Since nulliparous heifers have not been observed to be infectious in previous studies [19,20], the infectious class is neglected from the nulliparous compartment. Thus, the sub-populations considered by the model are $S_{np}$, $E_{np}$ and $A_{np}$ for the nulliparous compartment and $S_{mp}$, $E_{mp}$, $A_{mp}$ and $I_{mp}$ for the multiparous one.

Both compartments follow similar infection cycles. After the susceptible cattle ($S_{np}$ or $S_{mp}$) come in contact with *C. burnetii*, they immediately become exposed ($E_{np}$ or $E_{mp}$), with transmission rates depending on the susceptible sub-populations and on the indoor $L^{i}$ and outdoor $L^{o}$ environment compartments. Due to the different levels of contamination, each landscape of the environmental compartments contributes differently to the transmission of the bacteria; the detailed expression of the transmission rate is provided in the related to environment Section 2.1.3. For now, assume that the transmission rate follows a general frequency dependent form depending on the total indoor and outdoor environment transmission constants $\beta_{c}^{i}$ and $\beta_{c}^{o}$, respectively. In addition, the nulliparous cattle do not enter the main herd until their first lactation cycle, so that their risk of exposure is much lesser than that of multiparous cattle. This is incorporated by our model via the parameter γ<1, which only affects the transmission rate of nulliparous heifers.

After the cattle becomes exposed to the bacterium, they can either eliminate the disease or become infected. We incorporate the first event to the model by allowing the exposed cattle $E_{np/mp}$ to return to their previous susceptible state $S_{np/mp}$ at an elimination rate $\rho E_{np/mp}$, the rate constant of which is obtained by [7,12]. In the event when the exposed cattle $E_{np/mp}$ do not eliminate the disease, they move towards the asymptomatic state $A_{np/mp}$. This transition occurs at a rate $\alpha E_{np/mp}$, where the rate constant α expresses the seroconversion rate from initial exposure, which is assumed to be slightly slower than that reported for goats [21]. After being asymptomatic, the probability of a cattle losing its antibodies is considered negligible, especially in chronically infected herds, so that the transition from $A_{np/mp}$ to $E_{np/mp}$ is not allowed in our model.

The infected cattle eventually begin shedding into the landscape, a point at which they become infectious. However, since the shedding is intermittent [22], the infectious cattle are also allowed to return to their asymptomatic state, indicating that they temporarily stop shedding. Thus, our model accounts for the transitions from $A_{mp}$ to $I_{mp}$ and vice versa, but only for the multiparous compartment, since the infectious state is not considered for the nulliparous one. The transition from $A_{mp}$ to $I_{mp}$ is formulated by the rate $\tau A_{mp}$, while the one from $I_{mp}$ to $A_{mp}$ is formulated by the rate $\sigma I_{mp}$. The rate constants τ and σ were estimated by combining reported values in the literature [7,12] and *field* studies about the ratio of shedders over non-shedders [23]; see Appendix A. Finally, a removal pathway acting as a control measure is considered for the infected cattle $I_{mp}$. The removal rate $cI_{mp}$ represents herd management techniques (i.e., culling and isolation) and its rate constant *c* was adopted by [7], effectively corresponding in a 35% per annum culling rate.

The transitions related to the infection cycle of Q fever in both the multiparous and nulliparous compartment are denoted with solid arrows in the flow representation of Figure 1.

#### 2.1.2. Farm Demographics

The natural course of the Q fever infection is similar for the health status sub-populations of the nulliparous and multiparous compartments. Their interconnection occurs after the event of the first parturition, when a nulliparous heifer becomes a multiparous cow. This progression depends on the heifer’s current health status, so that the susceptible $S_{np}$, exposed $E_{np}$ and asymptomatic $A_{np}$ nulliparous heifers progress into the respective $S_{mp}$, $E_{mp}$ and $A_{mp}$ multiparous sub-populations. Our model considers these transitions to occur at a continuous rate, dependent on the rate constant δ, which essentially determines the percentage of multiparous and nulliparous cattle within the herd.

The general demographics of a herd are based upon the herd management practice of an individual farm. In the UK the prevalent practice is the all-year-round calving (AYR). For that herd management style, we assume that new cattle are introduced to the farm through two main pathways: livestock purchasing and births. A farm will purchase livestock for replacing the cattle that have been culled or isolated. The new cattle are assumed to be disease-free and thus, they are introduced to the susceptible multiparous class, $S_{mp}$. Regarding to births, we adopt a vertical disease transmission mechanism for our model. The new offsprings born from susceptible and exposed multiparous cows are assumed to be disease-free, so that they enter to the susceptible nulliparous sub-population $S_{np}$ at a birth rate $b(S_{mp}+E_{mp})$. However, the new calves born from asymptomatic and infectious multiparous cattle may be exposed to the disease. By considering this probability $p_{b}$, their offsprings enter the exposed nulliparous compartment $E_{np}$ at a birth rate $b\;p_{b}\left(A_{mp}+I_{mp}\right)$, while the rest enter the susceptible nulliparous compartment $S_{np}$ at a birth rate $b\;\left(1-p_{b}\right)\left(A_{mp}+I_{mp}\right)$. In addition, irrespectively of the herd management style, we assume that the old cattle are driven away from the herd through removal rates that are dependent on the sub-populations of the multiparous compartments. These removal pathways do not relate to isolation or culling due to infection by *C. burnetii* and occur at a rate constant μ for all multiparous sub-populations irrespectively of the health status of the infection cycle. All the aforementioned pathways related to the demographics of the herd are denoted with dashed black arrows in the flow representation of Figure 1.

In all-year-round calving (AYR), we assume that these practices (i.e., livestock purchasing and new offsprings birth) are performed in a continuous manner to replace the cattle that were removed from the herd. Thus, in order to represent a farm that actively manages the herd size, we impose in our model the restriction of maintaining the total cattle population constant. This restriction implies that (i) livestock purchasing is formulated in $S_{mp}$ by the same rate with cattle removal from the infectious compartment, $cI_{mp}$ and (ii) the farm removal rate μ is set equal to the birth rate *b*.

The justification for the selection of the various rate constants related to farm demographics is provided in Appendix A.

#### 2.1.3. Contamination of the Environment

New infections among the sub-populations are induced by infectious cattle $I_{mp}$ shedding *C. burnetii* through various shedding pathways; parturition products, faeces, urine, vaginal mucus or milk [23,24,25]. In the UK calves drink pasteurised milk, so that milk is excluded as possible route of infection. We model all the other possible infection mechanisms by assuming that infectious cattle contaminate a percentage of the total non-contaminated environment per day. The susceptible cattle then contract the disease from the contaminated environment, either through direct contact with shedding material or through inhalation of aerosolised particles.

Being particularly interested in examining the effect of different farm management techniques (continuous versus seasonal housing), the model explicitly distinguishes between a farm’s indoor and outdoor environments. This distinction is appropriate, since in the UK cattle normally undergo parturition in a separate building for various reasons, one being to mitigate the spread of disease throughout the herd. In addition, contamination of the indoor cattle housing micro-environment occurs indirectly, via both cattle and humans transporting contaminated material to the housing area through movements between the micro-environments. Thus, contamination from birthing materials is only considered for modelling the indoor environment.

**Figure 2:**
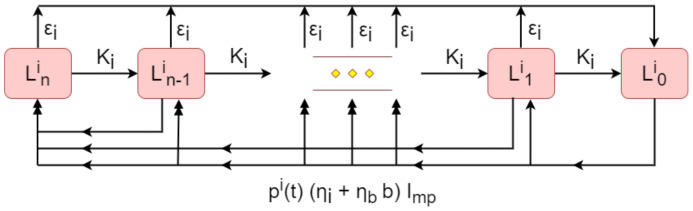
Flow diagram representation of the indoor environment. The multiple stages of contamination $L_{j}^{i}$ for j=0,⋯,n are denoted by round squares, which are connected by the black solid arrows, indicating the incremental decay of contamination, environmental hygiene and total indoor shedding output of the herd. The double arrows denote the accumulated contamination input to the $L_{j}^{i}$ stage by all less contaminated stages $L_{0}^{i},\ldots ,L_{j-1}^{i}$.

As illustrated in the flow diagram of Figure 2, the indoor environment is comprised of multiple stages of landscape contamination $L^{i}$. Each stage $L_{j}^{i}$ for j=0,⋯,n reflects the different levels of infectivity, due to the incremental decay of *C. burnetii*. The stage $L_{n}^{i}$ is considered the most contaminated and each subsequent landscape $L_{j}^{i}$ is less infectious than the last $L_{j+1}^{i}$, while the final stage $L_{0}^{i}$ is free from contamination. The purpose of the use of multiple stages that describe the landscape contamination is to account for the spatial heterogeneity of the bacterial distribution, i.e., some areas in the physical space may be more contaminated than others.

The infection by *C. burnetii* is introduced to the landscape by the total indoor shedding output of the herd. All stages $L_{j}^{i}$, except from the fully contaminated one, become more contaminated when shedding occurs. This transition is uniformly distributed along every stage of higher contamination $L_{k}^{i}$ for k>j,j≠0. Thus, after shedding, each stage can become more contaminated (hence more infectious) with contribution from all the lesser contaminated stages. Concretely, the proposed setup allows shedding to occur at any part of the landscape (except for the most contaminated one $L_{n}^{i}$). Hence, when a cow sheds in stage $L_{k}^{i}$ (j≠n), part of that landscape will become more contaminated. In order to account for the variability in the shedding output, e.g., whether the shedding output will increase that part of the landscape by one, two or more contamination levels, in the model we consider that the part of the landscape where the shedding occurs transitions to equally distributed parts in all higher contamination stages.

The shedding rate of the stage $L_{j}^{i}$ is $p^{i}\left(t\right)\left(\eta_{i}I_{mp}+\eta_{b}bI_{mp}\right)L_{j}^{i}$, which combines both the shedding rate $\eta_{i}$ from infectious cattle $I_{mp}$, and the shedding resulting from birthing products $\eta_{b}$, depending on the birth rate provided by the infectious cattle $bI_{mp}$. The indoor shedding rate constant $\eta_{i}$ was directly adopted from [7,12,17], while the birth shedding rate constant $\eta_{b}$ was estimated to match the ratio in [16] for the excretion of the bacterium from partus in comparison to that from faeces. Clearly, the contamination of the environment is enabled only when the herd comes in contact with the landscape, which is captured by the time-dependent parameter $p^{i}\left(t\right)$ representing the proportion of time per year during which the herd remains indoors.

As the contaminating bacteria within the landscape $L_{n}^{i}$ begin to naturally decay, a proportion of the landscape $K_{i}$ is transferred to the next landscape stage of lesser contamination $L_{n-1}^{i},L_{n-2}^{i},\ldots ,L_{1}^{i}$, up to the contamination-free stage $L_{0}^{i}$. This behaviour is modelled via the natural indoor environment decay rate $K_{i}L_{j}^{i}$, the rate constant of which was extrapolated from [17], where only one contaminated landscape was considered. In addition to this, we have added a control parameter $\epsilon_{i}$ that represents the environmental hygiene, which is the active removal of bacteria from the environment by farms (e.g., removing placenta discharge). The environmental hygiene clears part of each landscape stage $L_{j}^{i}$ for j=1,⋯,n at a rate $\epsilon_{i}L_{j}^{i}$, so that the $\sum_{j=1}^{n}\epsilon_{i}L_{j}^{i}$ is continuously becoming free from contamination, thus adding to $L_{0}^{i}$ stage. The active clearance rate constant $\epsilon_{i}$ was directly obtained by [17].

Regarding to the outdoor environment, similar landscape $L_{j}^{o}$ structuring was considered in our model. In this case however, the total outdoor shedding output of the herd is $p^{o}\left(t\right)\left(\eta_{o}I_{mp}\right)L_{j}^{o}$, since the term related to shedding from birthing products does not contribute to outdoor landscape contamination. Again, the contamination of the outdoor environment is introduced only when the herd remains outdoors, which is captured by the time-dependent parameter $p^{o}\left(t\right)$. In addition, no outdoor active clearing mechanism was considered, since such practices do not apply to the outdoor environment. In other respects, the outdoor environment operates similarly to the indoor environment. As a result, the respective outdoor shedding rate and natural decay rate, $\eta_{o}$ and $K_{o}$, respectively, are assumed to have the same values with the ones of the indoors environment.

The contract of the disease from the contaminated environment to the herd was assumed to follow a general frequency dependant transmission rate. Here, the transmission mechanism from susceptible to exposed cattle is revisited. Given the different levels of infectivity of each landscape, the probability of cattle to come in contact with shedding material depends on the contamination level of the landscape $L_{j}^{i}$ and $L_{j}^{o}$; in particular, the more contaminated the landscape, the greater the probability to contract the bacterium. In order to incorporate this mechanism in our model, the transmission rate was assumed to follow the expression $S\cdot f(L_{j}^{i},L_{j}^{o};t)$, in which:

(1) $$f\left(L_{j}^{i},L_{j}^{o};t\right)=p^{i}\left(t\right)\sum_{j=1}^{n}\beta_{j}^{i}L_{j}^{i}+p^{o}\left(t\right)\sum_{j=1}^{n}\beta_{j}^{o}L_{j}^{o}$$

where the parameters $p^{i}\left(t\right)$ and $p^{o}\left(t\right)$ represent the proportion of time per year during which the cattle remain indoors and outdoor, respectively, and the parameters $\beta_{j}^{i}$ and $\beta_{j}^{o}$ represent the transmission rate constants of each indoor and outdoor contaminated landscape $L_{j}^{i}$ and $L_{j}^{o}$, respectively, for j=1,…,n. We explicitly denote the dependency of the transmission rate on time, for highlighting that Equation (1) accommodates for different time-dependant housing styles (continuous vs. seasonal). However, due to the limited availability of experimental data, the calibration of the 2n parameters in Equation (1) is unfeasible without assumptions on the housing style and the level of infectivity of each contaminated landscape. We further discuss these matters after presenting the differential equations of the model.

### 2.2. The Mathematical Formulation of the Model

The *C. burnetii* within-herd transmission model is formulated by coupling the infection cycle of the nulliparous and multiparous sub-populations discussed in Section 2.1.1, with the effects provided by farm demographics and environment, as discussed in Section 2.1.2 and Section 2.1.3, respectively. Considering the sub-populations of different health status to be expressed as fractions over the total cattle population, the resulting differential equations for the nulliparous heifers are:

(2) $$\begin{array}{cc} \frac{dS_{np}}{dt} & =-\gamma S_{np}f\left(L_{j}^{i},L_{j}^{o};t\right)+\rho E_{np}-\delta S_{np}+b\left(S_{mp}+E_{mp}\right)+b\left(1-p_{b}\right)\left(A_{mp}+I_{mp}\right)\\ \frac{dE_{np}}{dt} & =\gamma S_{np}f\left(L_{j}^{i},L_{j}^{o};t\right)-\left(\rho +\delta +\alpha \right)E_{np}+bp_{b}\left(A_{mp}+I_{mp}\right)\\ \frac{dA_{np}}{dt} & =\alpha E_{np}-\delta A_{np} \end{array}$$

and for the multiparous cows are:

(3) $$\begin{array}{c} \begin{array}{cc} \frac{dS_{mp}}{dt} & =-S_{mp}f\left(L_{j}^{i},L_{j}^{o};t\right)+\rho E_{mp}+\delta S_{np}+cI_{mp}-\mu S_{mp}\\ \frac{dE_{mp}}{dt} & =S_{mp}f\left(L_{j}^{i},L_{j}^{o};t\right)+\delta E_{np}-\left(\rho +\alpha +\mu \right)E_{mp}\\ \frac{dA_{mp}}{dt} & =\alpha E_{mp}+\delta A_{np}+\sigma I_{mp}-\left(\tau +\mu \right)A_{mp}\\ \frac{dI_{mp}}{dt} & =\tau A_{mp}-\left(\sigma +c+\mu \right)I_{mp} \end{array} \end{array}$$

where the term $f(L_{j}^{i},L_{j}^{o};t)$ expresses the dependency of the transmission rates from the environment in Equation (1). Note that the restriction made in Section 2.1.2 about the birth rate *b* being equal to the removal rate μ implies that the sum of all the differential equations in Equations (2) and (3) equals to zero. This restriction further implies that the total cattle population remains constant:

$$S_{np}+E_{np}+A_{np}+S_{mp}+E_{mp}+A_{mp}+I_{mp}=1$$

expressing essentially the active management of the herd size.

With regards to the landscape contamination, the differential equations for the multiple stages of the indoors environment are:

(4) $$\begin{array}{cc} \frac{dL_{n}^{i}}{dt} & =p^{i}\left(t\right)\left(\eta_{i}+\eta_{b}b\right)I_{mp}\sum_{k=0}^{n-1}L_{k}^{i}-\left(K_{i}+\epsilon_{i}\right)L_{n}^{i}\\ \frac{dL_{j}^{i}}{dt} & =K_{i}L_{j+1}^{i}-\left(K_{i}+\epsilon_{i}\right)L_{j}^{i}+p^{i}\left(t\right)\left(\eta_{i}+\eta_{b}b\right)I_{mp}\left(\sum_{k=0}^{j-1}L_{k}^{i}-\left(n-j-1\right)L_{j}^{i}\right)\\ \frac{dL_{0}^{i}}{dt} & =K_{i}L_{1}^{i}+\epsilon_{i}\sum_{j=1}^{n}L_{j}^{i}-\left(n-1\right)p^{i}\left(t\right)\left(\eta_{i}+\eta_{b}b\right)I_{mp}L_{0}^{i} \end{array}$$

where j=1,…,n−1, and that of the outdoors environment are:

(5) $$\begin{array}{cc} \frac{dL_{n}^{o}}{dt} & =p^{o}\left(t\right)\eta_{o}I_{mp}\sum_{k=0}^{n-1}L_{k}^{o}-K_{o}L_{n}^{o}\\ \frac{dL_{j}^{o}}{dt} & =K_{o}L_{j+1}^{o}-K_{o}L_{j}^{o}+p^{o}\left(t\right)\eta_{o}I_{mp}\left(\sum_{k=0}^{j-1}L_{k}^{o}-\left(n-j-1\right)L_{j}^{o}\right)\\ \frac{dL_{0}^{o}}{dt} & =K_{o}L_{1}^{o}-\left(n-1\right)p^{o}\left(t\right)\eta_{o}I_{mp}L_{0}^{o} \end{array}$$

where j=1,…,n−1. Note that the stages $L_{j}^{i}$ and $L_{j}^{o}$ essentially express percentages of the total indoor and outdoor environment, thus their sum equals unity; i.e., $\sum_{j=0}^{n}L_{j}^{i}=1$ and $\sum_{j=0}^{n}L_{j}^{o}=1$.

The Q fever model in Equations (2)–(5) attains a disease-free and an endemic equilibrium, the derivation of which is presented in Appendix B. Since analytical expressions cannot be fully derived, we additionally report the numerical values of the equilibria.

#### 2.2.1. Model Parameterization and Parameter Reduction

The Q fever model in Equations (2)–(5) is composed of 7 sub-populations and 2*n* stages of landscape contamination; summing up to a total of 2n+7 variables. For its calibration, it is required to estimate 16+2n parameter values. Due to the high number of parameters (especially when considering high *n*) and due to the limited available literature and data for cattle herds in the UK, we followed a mixed approach. For the 8 parameters, for which the same to ours rate was utilised in other *in silico* studies, we directly adopted the corresponding parameter value. When no similar rate was available in the modelling literature, we calibrated the 5 parameter values according to related bacterium or infection characteristics, known from *in silico* studies, *field* studies or reports from official UK’s sources. Finally, we assumed the parameter values of γ, $\eta_{o}$ and $K_{o}$, for which no related studies were available in the literature. A detailed description on the estimation of the parameter values is provided in Appendix A. The rest 2n parameters are related to the transmission of the bacteria from the environment in Equation (1). Since the availability of data is limited, we incorporated two assumptions in the model for reducing the number of parameters to 6 in total, the 4 out of which can be estimated from the available literature.

First, the parameters $p^{i}\left(t\right)$ and $p^{o}\left(t\right)$ in Equations (1), (4) and (5) are determined in order to account for different housing styles. For continuous housing, $p^{i}\left(t\right)$ is set to one and $p^{o}\left(t\right)$ to zero, since the cattle remain indoors all year long. However, when accounting for seasonally housing, $p^{i}\left(t\right)$ and $p^{o}\left(t\right)$ change throughout the year. In order to estimate them, we assumed that cattle graze outdoors all day long and stay indoors during the night from March to September, while for the rest of the year they are housed for the winter. This assumption implies:

(6) $$p^{i}\left(t\right)=\left\{\begin{array}{cc} 0.5 & \mathrm{Mar}\leq t<\mathrm{Sep}\\ 1.0 & \mathrm{Sep}\leq t<\mathrm{Mar} \end{array}\right.\;p^{o}\left(t\right)=\left\{\begin{array}{cc} 0.5 & \mathrm{Mar}\leq t<\mathrm{Sep}\\ 0.0 & \mathrm{Sep}\leq t<\mathrm{Mar} \end{array}\right.$$

Secondly, in order to estimate the transmission rates $\beta_{j}^{i}$ in Equation (1), we assumed that the transmission probability from the contaminated landscape $L_{j}^{i}$ is lesser by a fractional reduction rate $r_{i}$ than the transmission probability from the immediately lesser contaminated landscape $L_{j-1}^{i}$. This assumption allows for estimating only the two parameters $r_{i}$ and $\beta_{n}^{i}$, rather than the n−1 in number $\beta_{j}^{i}$ transmission rates, since $\beta_{j}^{i}=r_{i}\beta_{j-1}^{i}={\left(r_{i}\right)}^{n-j}\beta_{n}^{i}$ for the indoor environment and $\beta_{j}^{o}=r_{o}\beta_{j-1}^{o}={\left(r_{o}\right)}^{n-j}\beta_{n}^{o}$ for the outdoor environments. Thus, given the total transmission rate constants, $\beta_{c}^{i}$ and $\beta_{c}^{o}$, and the fractional reduction rates, $r_{i}$ and $r_{o}$, each contaminated compartment’s transmission rate can be calculated as:

(7) $$\beta_{j}^{i}={\left(r_{i}\right)}^{n-j}\frac{\beta_{c}^{i}}{\sum_{k=1}^{n}{\left(r_{i}\right)}^{n-k}}\;\beta_{j}^{o}={\left(r_{o}\right)}^{n-j}\frac{\beta_{c}^{o}}{\sum_{k=1}^{n}{\left(r_{o}\right)}^{n-k}}$$

for j=1,…,n. The total indoor transmission rate constant, $\beta_{c}^{i}$, was adopted from [17]. The total outdoor transmission rate constant was assumed to be proportional to the indoors one by a factor 0.01, which represents the ratio of the average available space that cattle has when housed indoors over the available space when grazing outdoors, according to data obtained from [26,27].

Following the above assumptions, the list of parameters utilised within the Q fever model in Equations (2)–(5) is summarised in Table 1, where the description, the value and the source of estimation is provided for each parameter.

For the simulation of the Q fever model in Equations (2)–(5), one needs to further determine the number of landscape contamination stages *n*. Considering a high number of stages allows for increased spatial heterogeneity of the bacterial distribution, albeit increasing the complexity of the model. In order to achieve the optimum balance, we initially considered 10 stages of landscape contamination per environmental compartment. We then reduced the number of stages one-by-one and tracked down the profile of the transmission rate in Equation (1) (results not shown). It was indicated that the minimum number of stages required for obtaining similar results is 5; thus, we used n=5 stages of landscape contamination per environmental compartment.

### 2.3. Initial Conditions

According to the latest report by the UK Department for Environment, Food and Rural Affairs (DEFRA) regarding to farming population [28], the percentage of nulliparous cattle (younger than 2 years old) is about 44%, while that of multiparous cattle is about 56%. Assuming the introduction of one infected cow to an 100 cattle herd, the initial conditions of the standard scenario are $S_{np}\left(0\right)=0.44$, $S_{mp}\left(0\right)=0.55$, $I_{mp}=0.01$ and the rest sub-populations set to zero. The environmental landscapes are all set to zero, except to the non-contaminated landscapes $L_{0}^{i}=L_{0}^{o}=1$. For simulating the standard case, the parameter set in Table 1 was utilized both for continuous ($p^{i}\left(t\right)=1.0$ and $p^{o}\left(t\right)=0.0$ throughout the year) and seasonal housing (varying $p^{i}\left(t\right)$ and $p^{o}\left(t\right)$ according to Equation (6)).

### 2.4. Sensitivity Analysis

In order to identify the parameters affecting the outcome of the model and quantify their impact, sensitivity analysis was performed for each parameter included in the model. Interested in prevalences, four different outcomes are considered after one and five years of initial herd infection, that are the prevalences of (i) seronegative cattle (SN: $S_{np}+E_{np}+S_{mp}+E_{mp}$), (ii) seropositive cattle (SP: $A_{np}+A_{mp}+I_{mp}$), (iii) seropositive non-shedding cattle (SP NS: $A_{np}+A_{mp}$) and (iv) seropositive shedding cattle (SP S: $I_{mp}$). The sensitivity of the model in Equations (2)–(5) to the 20 non-zero parameters shown in Table 1 was estimated through the *Sensitivity Index*:

(8) $$SI\left(p\right)=\frac{E_{out}\left(p_{0}+\delta p\right)-E_{out}\left(p_{0}\right)}{\delta p}\frac{p_{0}}{E_{out}\left(p_{0}\right)}$$

where $E_{out}$ denotes the prevalence on the basis of which the outcome of the model was calculated. Given a perturbation δp to the nominal parameter value $p_{0}$, the value of SI in Equation (8) provides a measurement of the relative model’s outcome to the change of the parameter *p*; for comparison purposes the perturbation is proportional to the parameter’s nominal value, such that $\delta p=0.2p_{0}$. Thus, a positive SI implies that an increase of the respective parameter would lead to an increase of the associated outcome while the opposite would stand for a negative index (i.e., a decrease of the parameter would lead to a decrease of the outcome).

## 3. Results

Following the development and calibration of the model in Equations (2)–(5), here the resulting behaviour of our model is assessed and comparison with the existing literature is carried out. For reinforcing the confidence level of the results, the confidence intervals of the solution profiles, as derived by the model using a wide parameter sampling, was also carried out; we refer the interested reader to Appendix C. In addition, the model parameters that have the larger impact to the outcome are identified through sensitivity analysis and the effect of the outdoors environment on the infection cycle is further investigated.

### 3.1. The Standard Case

**Figure 3:**
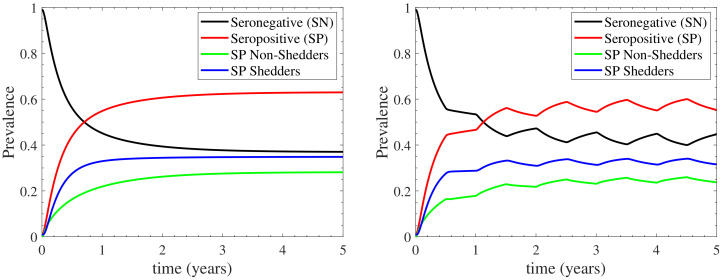
Prevalence of seronegative (SN, $S_{np}+E_{np}+S_{mp}+E_{mp}$) and seropositive (SP, $A_{np}+A_{mp}+I_{mp}$) cattle, with the seropositive ones being either non-shedders ($A_{np}+A_{mp}$) or shedders ($I_{mp}$). Both continuous (**left**) and seasonal (**right**) housing are considered; in the latter case the herd remains indoors/outdoors during the first/second half of each year.

We first present the results of the model in the standard case, which simulates the introduction of a 1% fraction of infected cows in a healthy herd. Figure 3 depicts the prevalence of seronegative and seropositive cattle, where the seropositive ones are either non-shedders or shedders; the left panel corresponds to continuous housing, while the right one to seasonal housing. In both cases, the long term behaviour of the model stabilises after the third year. In the continuous housing case, the prevalences stabilise in fixed values, which coincide to the endemic equilibrium values derived in Appendix B. In the seasonal housing case, the prevalences oscillate around fixed values, due to the seasonal activation of the indoors/outdoors environment. In particular, during the first half of each year (when the herd is housed only indoors), the prevalences tend to reach the endemic equilibrium values of the indoors case. However, during the second half of each year (when the herd grazes outdoors), the prevalences are driven to a lower endemic equilibrium, since the endemic equilibrium of the outdoors case is nearly disease-free as shown in Appendix B.

At the disease steady-state in both housing cases considered, as shown in Figure 3, the seronegative cattle constitute the 37% of the herd in continuous housing (∼42% in seasonal housing). Among the 63% of seropositive cattle, the 28% are non-shedding cattle while the 35% consist of shedding cattle (∼25% and ∼33% in seasonal housing, respectively). Note that, the confidence level of the aforementioned estimates is high, since the 95% confidence interval in the continuous housing case, shown in Appendix C, is recorded to deviate at most by only 0.6% from the mean values reported in Figure 3. The prevalences provided by our model are consistent with *in silico* studies reported in the existing literature. In particular, *in silico* studies of cattle herd in France reported 38% of seronegative non-shedding cattle and a 23% of seropositive non-shedding ones [12]. In addition, the prevalence of seropositive shedding cattle has been recorded to 39% [12], 32% [7] or 44% [14] by *in silico* studies. Although, the comparison to *in silico* data is generally not considered a good practice for the validation of a model, in the current case it can provide some reasonable confidence on the model’s reliability not only because of the scarcity of proper *field* data for dairy herds in the UK, but also because that the proposed model is structurally quite different from those used in the previous *in silico* studies. In addition to the aforementioned *in silico* studies, the proposed model demonstrates some fairly reasonable agreement with *field* studies that reported 46% [23] and 49% [18] prevalence of seropositive shedding cattle.

Further, our model additionally reproduces results from *in silico* studies regarding the temporal evolution of the standard case. In particular, the prevalence of seropositive cattle and seropositive shedding cattle follows the same temporal behavior with the one reported in [7]. According to Ref. [7], the prevalence of shedders reaches a steady-state value of ∼38% after the 1st year of infection. Similar behaviour is reported by our model in the standard case, as shown in Figure 3: the seropositive shedding cattle (blue curve) attain their endemic equilibrium value 35% just after the 1st year. The results reported in [7] indicate that the prevalence of seropositive cattle reaches their disease steady-state value slower than that of seropositive shedding cattle; a feature that is also reported in Figure 3. In addition, similar temporal evolution to the one displayed in Figure 3 has been reported to [13]. Finally, as reported in [33], when cows were imported into an area of endemic infection, 40% of uninfected cows became *C. burnetii* infected within 6 months. Our model again reproduces this temporal behaviour, since the seronegative cattle (black line in Figure 3) reduce to 60% up to the first 6 months of infection. In summary, our model is also able to reproduce findings from the existing literature with regard to the temporal evolution of the bacterium transmission.

#### 3.1.1. The Effect of Farm Demographics on the Infection Cycle

**Figure 4:**
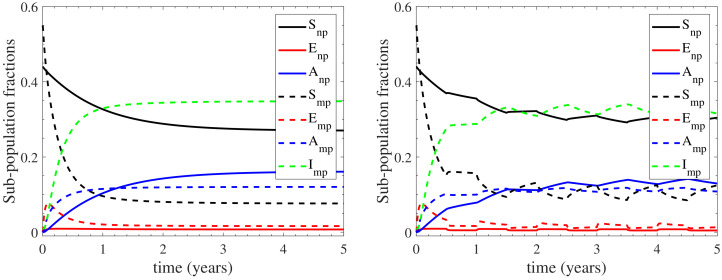
Evolution of the sub-populations profiles for the continuous (**left**) and seasonal (**right**) housing cases considered; in the latter case the herd remains indoors/outdoors during the first/second half of each year. The nulliparous sub-populations are depicted with sold curves, while the multiparous ones with dashed curves.

For assessing the effect of farm demography on the infection cycle, we demonstrate the temporal evolution of the sub-populations profiles in Figure 4 for both the continuous and the seasonal housing cases. We again highlight the high confidence level of the estimative in Figure 4, as reported by the confidence intervals for the continuous housing case in Appendix C, where the maximum deviation from the mean value is recorded to be at most 0.35%. It is shown that the nulliparous sub-populations follow qualitatively different infection dynamics in comparison to the multiparous sub-populations. In particular, $S_{np}$ faces a slow, gradual decrease during the first 3 years of infection and maintains a relatively high prevalence at ∼27% (∼30% in seasonal housing) thereafter. The resulting infection effect is almost wholly absorbed by $A_{np}$, which gradually increases for the first 3 years and stabilizes at ∼16% (∼13.5% in seasonal housing) thereafter, while $E_{np}$ is below 1% throughout the infection period. On the other hand, the multiparous sub-populations are more rapidly affecting the dynamics of the infection cycle. In particular, $S_{mp}$ reduces dramatically to ∼7.5% (∼10% in seasonal housing) during the first year of infection. This reduction is initially absorbed by the exposed sub-population $E_{mp}$ which acts as initial pool, since it rapidly increases at the very early times, at a rate higher than any other compartment. As a result, both the seropositive sub-populations $A_{mp}$ and $I_{mp}$ face a rapid increase, attaining a plateau at ∼12% and ∼35% respectively (∼11.5% and ∼33% in seasonal housing). It should be noted here that the sub-populations’ long-term response is expected to converge at the endemic equilibrium values reported in Appendix B. However, insights about dynamics of the infection spread due to farm demographics, such as the interesting role of $E_{mp}$ as an initial pool of infection, cannot be drawn from the endemic equilibrium.

In summary, the model’s response indicates the following regarding to farm demography: (i) the seronegative prevalence of the herd is mainly maintained by the nulliparous heifers (27% out of 37% in continuous housing and 30% out of 42% in seasonal housing) and to a much lesser degree by the multiparous cattle, (ii) both the nulliparous heifers and the multiparous cattle contribute almost to the same degree to the seropositive non-shedding prevalence of the herd; the multiparous sub-populations acting faster than the nulliparous ones, and (iii) the seropositive shedding prevalence, which only considers the infected state of the multiparous cattle, mainly originates from the low prevalence of the other multiparous sub-populations. In addition, the role of the exposed sub-populations should be highlighted: the exposed multiparous cattle initiate the infection cycle, while the nulliparous heifers do not stay at the exposed disease state for long periods; they either become susceptible or asymptomatic before becoming multiparous cattle. Finally, the slower effect of the nulliparous heifers to the infection cycle, in comparison to that of the multiparous cattle, can be explained by the common practice of housing pregnant heifers away from the main milking herd, where they will be exposed to relatively lower levels of contamination compared with the multiparous animals within the main milking herd.

#### 3.1.2. The Heterogeneity of the Environmental Contamination

**Figure 5:**
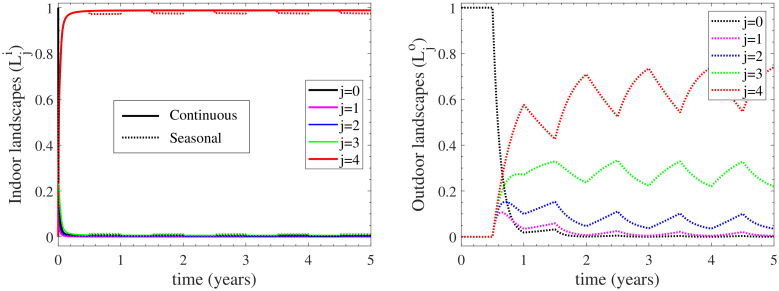
Environmental contamination to the infection spread provided by the landscapes $L_{j}^{i}$ and $L_{j}^{o}$ of the indoor (**left**) and outdoor (**right**) compartments, respectively. The contamination landscape in the continuous housing case is depicted with solid curves, while that of the seasonal housing is depicted with dotted curves; in the latter case the herd remains indoors/outdoors during the first/second half of each year. The outdoor environment is only activated in the seasonal housing case.

Since the outdoor environment is activated only when seasonal housing is considered, we first examine the heterogeneity of the indoor environment in the continuous housing case. The left panel of Figure 5 displays the temporal evolution of the indoor environmental contamination. It is reminded that $L_{0}^{i}$-$L_{4}^{i}$ and $L_{0}^{o}$-$L_{4}^{o}$ denote the (indoors and outdoors, respectively) contamination landscapes with $L_{0}^{i}$, $L_{0}^{o}$ being free of any contamination and $L_{4}^{i}$, $L_{4}^{o}$ representing the highest level of contamination. It is firstly shown that a rapid increase of the indoor most contaminated landscape is observed immediately after the introduction of the infection to the herd, since $L_{4}^{i}$ reaches the value of 0.95 at 0.3 years. In other words, 95% of the indoors environment reaches the highest level of contamination at 0.3 years; with the endemic equilibrium value being 97%, as shown in Appendix B. In addition, the landscape contamination is predominantly at the highest level, as this is manifested by the dominant value of $L_{4}^{i}$ and the negligible values of $L_{1}^{i}$-$L_{3}^{i}$. These results indicate that the indoors environment becomes very quickly contaminated at the highest level. Such a behaviour is not only attained at the endemic equilibrium, as expected by the steady-state values of the indoors landscapes $L^{i*}$ reported in Appendix B, but also throughout the dynamics of the infection spread.

Considering now the case of seasonal housing, only minor differences are reported for the indoor environmental load, as shown in the left panel of Figure 5. In particular, at the second half of each year, the most contaminated landscape $L_{4}^{i}$ slightly decreases; a result that is reflected in the minor increase of the contamination free compartment $L_{0}^{i}$ during this time of the year. This is because the cattle herd stays indoors only during the night when seasonal housing is considered, and grazes outdoors all day long. As a result, the outdoors environment is activated, as shown in the right panel of Figure 5. However, the outdoors environment follows different dynamics than the indoors one. The first striking difference relates to the rate of change of the contamination landscapes, which for the outdoors environment are generally much slower than the indoors ones; the most contaminated landscape $L_{4}^{o}$ needs half a year to reach 60% of the whole landscape, while the $L_{4}^{i}$ needs less than 0.3 years to reach 95% of the whole landscape. After that time, $L_{4}^{o}$ oscillates around the value of 65%. This behaviour also relates to the time needed for the contamination free compartment to reach its minimum, which for the indoors environment occurs almost immediately after infection, while for the outdoors one occurs after 1.8 years; see $L_{0}^{i}$ and $L_{0}^{o}$ levels. The second notable difference between the two environments is that the landscape contamination of the outdoors environment is distributed along several compartments, hence highlighting the heterogeneity of the contamination landscape, in contrast to the indoors environment where the contamination becomes in principle homogeneous. In particular, during the first year, when the most contaminated outdoors compartment $L_{4}^{o}$ reaches a maximum, the compartments representing lower contamination landscapes ($L_{1}^{o}$, $L_{2}^{o}$, $L_{3}^{o}$) become important. However, after the first couple of years, the first two contamination landscapes $L_{1}^{o}$ and $L_{2}^{o}$ become practically zero and the dominant ones remain $L_{4}^{o}$ and $L_{3}^{o}$ with some minimal also contribution from $L_{2}^{o}$. This long-term (oscillatory) behaviour is bounded by the endemic equilibria of the extreme cases of solely indoor and solely outdoor housing, derived in Appendix B.

In summary, the differences between the two environments in the seasonal housing case suggest that the contamination from the outdoors environment serves as a pool to preserve the levels of infection for a long period and potentially spread the disease to other herds. This is also supported by the fact that the sub-population of exposed cattle in the case of seasonal housing are maintained to higher levels in comparison to that of continuous housing, as shown in Figure 4. However, the viability of the organism will eventually reduce outdoors and when it does, indoor contamination dominates. From a modelling and biological perspective, the aforementioned differences between indoor and outdoor bacterial loads are primarily due to the relatively high levels of birth shedding rate $\eta_{b}$ in comparison with other primary routes (e.g., faecal, vaginal) [6].

A direct comparison of the previously discussed results with the existing literature is difficult to be performed, mainly for three reasons, all relating to the structure of the models used. Firstly, in the previous works (e.g., [7,16]) the environmental load is measured in *environmental units*, which is not the case in our model. Secondly, no distinction is made between indoors and outdoors, but rather the net environmental load is considered. Thirdly, unlike the current work, the compartmentalisation of the environment includes one compartment and does not account for the landscape heterogeneities.

### 3.2. Sensitivity Analysis

In order to assess the sensitivity of the model’s parameters on the model’s response, sensitivity analysis was performed for each of the 20 non-zero parameters incorporated in the model. The sensitivity index SI(p) of each parameter *p* was calculated according to Equation (8), given its nominal parameter value from Table 1. A high value of SI(p) indicates that a small change in the value of the parameter *p* will have a significant impact on the desired model’s outcome. This implies that the parameters that are determined as important by sensitivity analysis should be characterised by low uncertainty; the ones with high uncertainty should be more accurately determined. As a result, the purpose of this task is also to use the obtained results for the design of future experimental campaigns that will enable the accurate determination of the parameters identified with high sensitivity on the model’s response.

**Figure 6:**
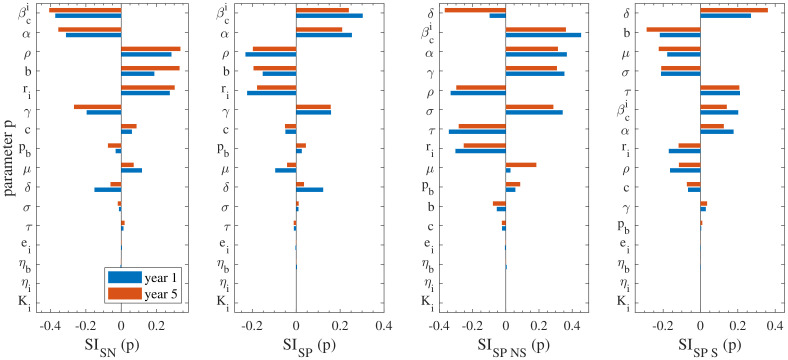
Visualised sensitivities SI of the model outputs for each parameter *p* calculated by Equation (8) after one and five years (blue and red bars, respectively), for the continuous housing case. The model outputs are the prevalences of seronegative, seropositive, seropositive non-shedding and seropositive shedding cattle and the related sensitivities $SI_{SN}$, $SI_{SP}$, $SI_{SP\;NS}$ and $SI_{SP\;S}$ are shown from the left panel to right one. The indices SI are sorted in descending order on the basis of the 5-year output.

Here, the model’s performance and response to the applied perturbations is investigated in view of the seronegative, seropositive, seropositive non-shedding and seropositive shedding cattle populations. We evaluate the response after one year post initial herd infection in order to examine the transition to the steady-state and at five years post initial herd infection when the system has reached its steady state. The results of this investigation for the continuous housing case are visualized in Figure 6, where all the parameters except those of the outdoors environment are considered. The results for the seasonal housing case (for which we considered an average of the fifth year post initial herd infection) are qualitatively similar to the continuous one; we report the exact SI(p) values for both cases in Supplementary Table S1.

Firstly, the analysis reveals that the most important parameter for most sub-populations is $\beta_{c}^{i}$, i.e., the total indoor transmission rate. This is an intuitive finding, since it is related to the source of infection, the transmission rates of $S_{np}/S_{mp}$ becoming $E_{np}/E_{mp}$. In particular, $\beta_{c}^{i}$ is the most important parameter for the seronegative and seropositive and second most important for seropositive non-shedders but less important for the seropositive shedders. The respective SI is negative for the seronegative sub-population and positive for the remaining seropositive ones. This is reasonable, since an increase of the total indoor transmission rate will obviously lead to increase of the seropositive and a decrease of the seronegative sub-populations. In the current work, we calibrated the value to match that in [12], in which multiple indoor compartments were not utilised. Thus, although there is some reasonable certainty in the use of this parameter, further experimental studies are required for reinforcing confidence in its usage.

The importance of α is highlighted next, that is the seroconversion rate, which expresses the time needed for cattle to become antibody positive after being exposed to the bacterium. This parameter is expected to play significant role (especially in seronegative, seropositive and seropositive nonshedders) and its uncertainty is relatively low, since it has been estimated in previous works (see, e.g., [21]). It is noted that it has negative sign for the seronegative sub-population and positive for the remaining ones, similar to $\beta_{c}^{i}$. This is an expected outcome because an increase of α indicates a decrease of the time needed for the cattle to become antibody positive, hence seropositive sub-populations would tend to increase.

The next most important parameter was determined to be ρ, that expresses the probability of an exposed cattle to eliminate the disease; thus transit to the susceptible sub-populations $S_{np}/S_{mp}$. Data available in the existing literature could be considered sufficient for the determination of this parameter with confidence. In the current study, this parameter was determined through the works of [7,12,17]. It is noted that the SI of ρ is positive only for the seronegative sub-population and negative for the remaining three (seropositive, seropositive non-shedders, seropositive shedders), thereby indicating that an increase of the parameter’s value would lead to an increase of the seronegative sub-population and decrease of the three remaining ones. Again, this is an expected outcome, since an increase in ρ indicates an increasing probability of the exposed cattle to eliminate the disease, hence seropositive prevalence would tend to increase.

The birthing rate *b* of multiparous cattle is also found to have some notable effect on the model’s performance. This parameter relates to demographics and generally has low uncertainty, since there are sufficient data in the literature. In the current study this parameter was calibrated on data from [7,29,30,31]. It should be highlighted though that both *b* and δ parameters are herd-dependant (they vary according to farm demographic techniques AYC, etc.). Figure 6 shows that an increase of *b* leads to the increase of the seronegative sub-populations and decrease of the seropositive ones. Its impact becomes particularly insignificant for the seropositive non-shedders.

Another parameter that merits separate discussion is δ, which expresses the nulliparous to multiparous progression rate. The sensitivity analysis highlighted that δ has particularly high sensitivity index for the seropositive non-shedders and shedders, especially at the long-term, and becomes much less important for the seropositive population as a total and the seronegative as well. This was an unexpected finding and in fact this analysis verified the importance of considering separately the nulliparous and the multiparous subpopulations from a modelling perspective. Thanks to sufficient demographics data in the literature [28], this parameter has been determined with a sufficient degree of certainty.

The following parameters were also identified through the sensitivity analysis to have some non-negligible impact on the model’s performance:

- $r_{i}$, that is, the reduction rate of transmission probability by the indoors environment, which essentially provides a measure of the uniformity of the transmission rate in the different environmental compartments: the higher the value of $r_{i}$ the more uniform the distribution of the transmission rates. There are currently no field or experimental data available in the literature for this parameter, therefore its uncertainty in the current study is high. Previous studies have not considered compartmentalisation of the environment, thus it is a new result highlighted by our model. Consequently, future experimental or field studies should focus on estimating this parameter. It is noted that the SI of $r_{i}$ is positive/negative for the seropositive/seronegative prevalence, suggesting that the more uniform the transmission rates become among the different environmental compartments, the more the seropositive sub-population.
- τ and σ, expressing the rates from $A_{mp}$ to $I_{mp}$ and vice versa, i.e., asymptomatic to infected. In the current study, data available from the literature were used to calibrate these parameters [7,12,23]; yet all the previous studies were performed in French cattle herds and did not distinguish between exposed and asymptomatic cattle. Therefore, there is some need for further research to enable a potentially more accurate description of these parameters.
- γ, that is the ratio of reduced transmission from nulliparous cattle. There is no data available in the literature for this parameter, hence, it is characterised by high uncertainty. As a result, further research is required to this regard. The importance of this parameter is also underlined by the fact that previous studies conclude that vaccination should be focused on nulliparous cattle [7,34,35];

The identification of $\beta_{c}^{i}$, $r_{i}$ and γ is an important finding because it underscores the key role that these parameters have at the model’s outcome. Their identification is fully justified because they all relate to the source of the infection, i.e., the mechanism through which the herd is infected by the environmental load. Results from previous works also highlight this importance [7,12,14,16].

The valuable, yet limited, insight that is obtained through the sensitivity analysis, also indicated another outcome related to the outdoors environment. The sensitivity of the seropositive and seronegative (both shedding and non-shedding) populations to all the parameters related to the outdoor environment ($r_{o}$, $b_{c}^{o}$, $\eta_{o}$ and $K_{o}$) is negligible; see the corresponding SI(p) for the seasonal housing case in Supplementary Table S1. This result would indicate the minor effect of the outdoors environment to the model’s response. However, this is not the case, as highlighted in the following Section.

### 3.3. The Effect of the Outdoors Environment on the Infection Cycle

In order to further assess the role of the outdoors environment, we examined a number of seasonal housing scenarios, where each of the factors affecting the dynamics of the outdoors environment was investigated. We highlight here that in the case where the cattle herd is solely housed outdoors, the Q fever infection nearly ebbs away, as indicated by the nearly disease-free equilibrium in Appendix B. Thus, examining seasonal housing cases of extended time spent outdoors would also provide insight for the regulation of the infection.

As expected by the sensitivity analysis results, no significant effect (data not shown) to the infection cycle was reported when considering significantly different (more than 10-fold increase/decrease) outdoor shedding rates $\eta_{o}$, natural environmental decay rates $K^{o}$ or different reduction rates of transmission probability $r_{o}$. The most significant effect, albeit minor, was reported (data not shown) when decreasing the available outdoor space of the cattle herd to graze. In the standard seasonal housing case considered in Section 3.1, the herd grazes outdoors in a landscape 100 times more spacious than the farm’s indoors building space (reflected in $\beta_{c}^{o}$ being the 1% of $\beta_{c}^{i}$ in Table 1). By considering a smaller farm scenario, where the outdoors landscape is only 5 times more spacious than the indoors space, indicated a 0.9% decrease of the seronegative sub-population and a 0.5% and a 0.4% increase of the seropositive shedding and non-shedding, respectively, sub-populations during the fifth year post initial herd infection. Clearly, the effect of the outdoor available space is minor for the dynamics of the infection.

**Figure 7:**
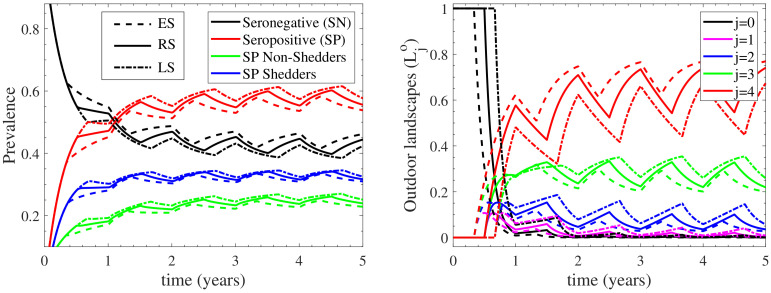
Comparison of the extended seasonal (ES) and limited seasonal (LS) housing cases with the regular seasonal (RS) case. The **left** panel depicts the prevalence of seronegative (SN) and seropositive (SP) cattle, accounting for non-shedders and shedders. The **right** panel shows the outdoor environmental load of the contaminated landscapes $L_{1,2,3,4}^{o}$ and the contamination free landscape $L_{0}^{o}$.

To demonstrate the role of the outdoors environment, two additional scenarios of different seasonal housing were considered. In the first “extended” seasonal (ES) case, the cattle herd is grazing outdoors for an extended period of 8 months (February to October), while in the second, “limited” seasonal (LS) case, the cattle herd is now grazing outdoor for a limited period of 4 months (April to August). The model’s response in these two cases was compared to the regular seasonal (RS) case in Figure 7, for which outdoor grazing lasts for 6 months. As shown in the left panel of Figure 7, the seronegative sub-population in the ES case is increased in comparison to that of the RS case, which is also higher than that of the LS case; the inverse effect is depicted for the seropositive (both shedding and non-shedding) sub-populations. In addition, as shown in the right panel of Figure 7, the most contaminated landscape of the outdoor environment $L_{4}^{o}$ in the ES case is more contaminated when compared to the RS case, which in turn is also more contaminated than that of the LS case. As a result, the less contaminated stages $L_{1,2,3}^{o}$ attain decreasing contamination levels when comparing the ES, RS and LS cases. These results indicate that as the period during which the cattle herd remains outdoors becomes extended, the prevalence of seropositive cattle decreases, despite the fact that the most contaminated landscape of the outdoor environment is more contaminated. This occurs because when the outdoors housing is extended, the respective indoors shrinks, hence the influence of the indoor transmission rate (which is 100 times larger than the outdoor transmission rate) becomes less effective.

In summary, the outdoors environment acts as an indirect suppressor of the infection dynamics, since (i) when altering the parameters of the model related to the outdoors environment, no significant effect to the infection cycle is reported, while (ii) when accounting for extended periods spent by the cattle herd outdoors, significant decrease of the seropositive cattle is reported.

## 4. Conclusions

The epidemiological Q fever transmission model presented in this work interconnects the within-herd infection cycle of *C. burnetii* with the effect of parturition, farm demographics and both indoor and outdoor environment contamination. Due to the limited availability of experimental and field data, the model was developed to include a minimal number of parameters; this implied the assumptions of constant total cattle population and linearly distributed transmission probabilities of *C. burnetii* by the indoor and outdoor environmental landscapes. The model was calibrated with parameter values from the existing literature and available dairy cattle herd demographics data obtained by the UK DEFRA and other UK’s sources [28,30,31].

The findings presented in Section 3.1 indicate consistency of the results obtained by the proposed model against *in silico* studies as well as some reasonable agreement with field studies, both regarding the disease steady-state of the cattle herd and the temporal evolution of the bacterium transmission. Our results further highlight the important role of the indoor and outdoor environmental contamination. The former dominates the transmission route, while the latter one serves as a pool to preserve high levels of infection for longer periods. Given the increased survival of the bacterium in adverse environmental conditions [36], the latter result suggests the importance of the outdoor environment on chronically infected herds and disease spread towards other herds as well. The sensitivity analysis of the model in Section 3.2 suggested the direction for future experimental and *field* studies for providing better understanding of the disease dynamics. In particular, the parameters identified with high sensitivity and increased uncertainty (the ones with low uncertainty are sufficiently determined) relate to the transmission mechanism of the bacterium from environmental compartments with different degree of contamination.

Finally, the investigation of different housing techniques (demonstrated to some degree in Section 3.1 and Section 3.3) and farm management styles is enabled by the model presented in this work, due to its inclusive character. In addition, the model can accommodate the consideration of a variety of interventions targeted specifically to the indoor and/or outdoor environments (ventilation, active clearance); thus, serving as a modelling proof for their assessment. For example, as indicated in Section 3.3, the outdoors environment acts as an indirect suppressor of the infection dynamics; hence, one interventional approach would be to adopt a housing style with extended outdoor grazing.

## Figures and Tables

**Table 1:** The set of parameters and their description considered for the development of the model in Equations (2)–(5). CtM: Calibrated to Match.

Parameter		Unit	Description	Source
$p^{i}$, $p^{o}$		-	Proportion of time per year for which cattle stay indoors and outdoors	Equation (6)
$\beta_{j}^{i}$, $\beta_{j}^{o}$		day^−1^	Transmission rates of $S_{np}/S_{mp}$ becoming $E_{np}/E_{mp}$ from each indoor and outdoor contaminated landscape	Equation (7)
$r_{i}$, $r_{o}$	0.8	-	Reduction rate of transmission probability by indoor and outdoor environment	Assumed
$\beta_{c}^{i}$	0.0943	day^−1^	Total indoor transmission rate	[12,17]
$\beta_{c}^{o}$	$0.01\beta_{c}^{i}$	day^−1^	Total outdoor transmission rate	CtM [12,26,27]
γ	0.1	-	Ratio of reduced transmission rate for nulliparous cattle	Assumed
ρ	0.1	day^−1^	Transition rate of $E_{np}/E_{mp}$ eliminating the disease	[7,12]
α	0.04762	day^−1^	Transition rate of $E_{np}/E_{mp}$ becoming $A_{np}/A_{mp}$	[21]
τ	0.09023	day^−1^	Transition rate of $A_{mp}$ becoming $I_{mp}$	CtM [7,12,23]
σ	0.02857	day^−1^	Transition rate of $I_{mp}$ becoming $A_{mp}$	[7,12]
*c*	$9.57\times 10^{-4}$	day^−1^	Removal rate of $I_{mp}$ due to culling and isolation	[7]
δ	0.0021	day^−1^	Progression rate from nulliparous to multiparous cattle	CtM [28]
*b*	1/600	day^−1^	Birth rate of multiparous cattle	[7,29,30,31]
$p_{b}$	0.3	-	Probability of the offspring being exposed after birth from $A_{mp}/I_{mp}$	[7,32]
μ	*b*	day^−1^	Natural death and removal rate	CtM [7,12,17]
$\eta_{i}$	0.04	day^−1^	Indoor shedding rate from $I_{mp}$	[7,12,17]
$\eta_{b}$	$\eta_{i}/2.7\times 10^{-6}$	-	Indoor birth shedding rate from $I_{mp}$	CtM [16]
$\eta_{o}$	0.04	day^−1^	Outdoor shedding rate from $I_{mp}$	Assumed
$K_{i}$	0.0083/n	day^−1^	Natural indoor environment decay rate	CtM [17]
$K_{o}$	0.0083/n	day^−1^	Natural outdoor environment decay rate	Assumed
$\epsilon_{i}$	0.1	day^−1^	Active clearing rate of contaminated indoor environment	[17]

**Figure A1:**
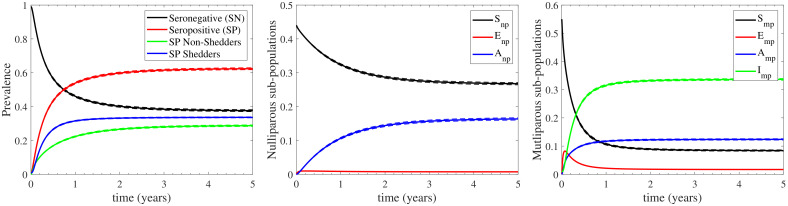
Prevalence (**left panel**) of seronegative (SN) and seropositive (SP) cattle for the continuous housing case, as in the left panel of Figure 3. Nulliparous (**middle panel**) and multiparous (**right panel**) sub-population profiles for the continuous housing case, as in the left panel of Figure 4. The solution profiles are derived on the basis of the 1000 parameter sets considered, with the mean values shown in solid and the 95% confidence intervals in dashed.

## Data Availability

Not applicable.

## Supplementary Materials

The following supporting information can be downloaded at: https://www.mdpi.com/article/10.3390/vetsci9100522/s1.

## Appendix A. Parameterization of the Model

For the parameterization of the model in Equations (1)–(5), due to the scarce available literature and the limited data for cattle herds in the UK, we followed a mixed approach. For the cases where the same transmission/transition/removal/etc. rate was utilised in other in silico studies, the corresponding value of the parameter was directly adopted. When no similar rate was available in the modelling literature, we calibrated its parameter value to match related bacterium or infection characteristics, known from in silico studies, *field* studies or reports from official UK’s sources. When no related studies were available to parameterize a specific rate, we assumed the related parameter value, so that the model approximately reproduces quantitative results from other studies. In order to minimize the tuned parameters, we also imposed the assumptions described in Section 2.2.1. In the following, we present the derivation of each parameter value shown in Table 1.

### Appendix A.1. Transmission Related Parameters

As discussed in Section 2.1.1 and Section 2.1.3 the susceptible cattle of the herd contract the disease from the contaminated landscapes $L_{j}^{i}$ and $L_{j}^{o}$, either through direct contact with shedding material or through inhalation of aerosolised particles. The overall transmission rate is modelled by the expression $S_{mp}f\left(L_{j}^{i},L_{j}^{o};t\right)$ for the multiparous cattle. The reduced environmental transmission of the nulliparous heifers is modelled by the overall transmission rate $\gamma S_{np}f\left(L_{j}^{i},L_{j}^{o};t\right)$. The parameter γ expresses the reduced probability of transmission to nulliparous heifer in comparison to that of the multiparous cattle and it was tuned to 10%, since no data are available in the literature for this parameter. In addition, the expression of $f(L_{j}^{i},L_{j}^{o};t)$ in Equation (1) includes 2n parameters. The 2 parameters $p^{i}\left(t\right)$ and $p^{o}\left(t\right)$ represent the proportion of time per year during which the cattle remain indoors and outdoors, respectively; their values were realistically assumed in Equation (6) for modelling continuous and multiple seasonal housing techniques. The rest 2n−2 parameters in Equation (1), $\beta_{j}^{i}$ and $\beta_{j}^{o}$, represent the transmission rate constants of each indoor and outdoor contaminated landscape $L_{j}^{i}$ and $L_{j}^{o}$, respectively, for j=1,…,n. In order to avoid overparameterization and since similar transmission rates exist in the literature for only one environmental compartment, we introduced the fractional reduction law in Equation (7) by assuming that the transmission probability from each contaminated landscape is lesser, by a fraction reduction term $r_{i}$ and $r_{o}$, than the transmission probability from the immediately lesser contaminated landscape. Using Equation (7) the number of parameters is reduced from 2n−2 to 4; the total indoor transmission rate constants, $\beta_{c}^{i}$ and $\beta_{c}^{o}$, and the fractional reduction terms $r_{i}$ and $r_{o}$. The total indoor transmission rate constant $\beta_{c}^{i}=0.0943$ day^−1^ was directly adopted from [17], who derived the parameter value from in days from [12]. The total outdoor transmission rate constant was calibrated to be proportional to the indoors one, so that $\beta_{c}^{o}=0.01\beta_{c}^{i}$; the factor 0.01 represents the ratio of the average available space that a cattle has when housed indoors (mean of 8.3 m^2^ per cattle [26]) over the available space when grazing outdoors (average pasture area of 1004 m^2^ per cattle [27]). The fractional reduction terms $r_{i}$ and $r_{o}$ were tuned to 0.8, since no data are available in the literature for this parameter. Tuning the values $r_{i}$ and $r_{o}$ depends on the number of stages *n* of landscape contamination. As also discussed in Section 2.2.1, we started with n=10 and reduced *n* one-by-one until reaching n=5, tuning $r_{i}$ and $r_{o}$ so as the profile of the overall transmission rate $f(L_{j}^{i},L_{j}^{o};t)$ in Equation (1) does not vary much quantitively.

### Appendix A.2. Infection Cycle Related Parameters

According to the discussion in Section 2.1.1, the model considers 4 (3) health states for the multiparous (nulliparous) compartment. Susceptible cattle become exposed via the transmission rates discussed in Appendix A.1. Exposed cattle can return to their susceptible state with an elimination rate $\rho E_{np/mp}$. Since the exposed cattle do not have yet developed antibodies, we adopted directly the rate constant ρ=0.1 from [7,12] (was also used in [17]), where the rate constant was used to formulate the transition rate from exposed cattle without antibodies to the susceptible ones. The transition of exposed cattle to the asymptomatic state (infected but not shedding) is modelled by the rate $\alpha E_{np/mp}$, where the parameter α is the seroconversion rate from initial exposure. For this parameter, we adopted directly the value α=1/21 day^−1^, since approximately 3 weeks are needed for a cattle to become antibody positive (slightly longer than the period reported for goats [21]). The transition from the asymptomatic to the infectious state and vice versa (due to intermittent shedding [22]) is modelled only for multiparous cattle, with the transition rates $\tau A_{mp}$ and $\sigma I_{mp}$, respectively. For determining σ, we directly adopted the value 0.2 per week from [7,12], which therein expresses the transition rate constant from shedding cows with antibodies to non-shedding cows with antibodies. However, since in [7,12] there are more than one shedding states, the reverse transition rate constant from non-shedding cows with antibodies to shedding ones (mean of 0.1286 per day) is not an appropriate selection for τ, herein. Instead, we calibrated τ to match the data from the *field* study [23], where the ratio of shedders over non-shedders is 60/19 (see Table VIII therein for the seropositive cows on a weekly basis); thus deriving the value of τ=60/19σ=0.09023 per day. This value is smaller, but very close to the one considered in [7,12]. Finally, a removal pathway is considered for the multiparous infected cattle $I_{mp}$, through the removal rate $cI_{mp}$. We directly adopted the culling rate constant $c=9.57\cdot 10^{-4}$ from [7] (see the average of culling rates in Table 2 therein), effectively corresponding to a 35% per annum culling rate.

### Appendix A.3. Farm Demography Related Parameters

Since we are interested in modelling the cattle herds in the UK, in order to parameterize the model, we considered available data from the latest official UK’s sources reports [28,29,30,31]. After the event of parturition, the multiparous cattle give birth to newborn offsprings, which are considered in the nulliparous sub-population in our model, either being disease-free or exposed during parturition [5,6]. In particular, as also discussed in Section 2.1.2, the new offsprings born from susceptible and exposed multiparous cows are assumed to be disease-free and enter the susceptible nulliparous sub-population $S_{np}$ at a birth rate $b(S_{mp}+E_{mp})$. However, the new calves born from asymptomatic and infectious multiparous cattle may be exposed to the disease. By considering this probability $p_{b}$, their offsprings enter the exposed nulliparous compartment $E_{np}$ at a birth rate $b\;p_{b}\left(A_{mp}+I_{mp}\right)$, while the rest enter the susceptible nulliparous compartment $S_{np}$ at a birth rate $b\;\left(1-p_{b}\right)\left(A_{mp}+I_{mp}\right)$. In order to determine the birth rate constant *b*, we considered the a 400 days calving interval in a standard UK dairy herd according to the reports [29,30]. However, according to the latest news and the projections of the Agriculture and Horticulture Development Board (AHDB) [31], the female-male birth ratio is estimated to 2/3. Since we are interested in only female births, the birth rate constant was estimated to b=1/600day^−1^; a parameter value that is in agreement with the total calving interval (calving interval, dry period and non-gestation period for French cattle) in [7]. In addition, we assumed that the probability of the offspring being exposed to the bacterium after birth is 30% (i.e., $p_{b}=0.3$), which is in agreement with the vaginal shedding parturition probabilities reported in [7,32]. Further, we modelled the removal rates of each multiparous cattle sub-population using the same rate constant μ, which represents removal from the herd due to age and not isolation or culling (which is incorporated in the parameter value of *c*). As discussed in Section 2.1.2, in order to impose in our model the restriction of maintaining the total cattle population constant, it is implied that the farm removal rate μ is set equal to the birth rate *b*. Although this parameter is calibrated indirectly to our model, its value μ=1/600day^−1^ agrees with the natural death rate estimated in [7,12,17], summed up with a removal rate corresponding to cows over 6 years old being removed from the herd. Finally, we assumed that after the event of the first parturition, a nulliparous heifer becomes a multiparous cow and modelled this progression to occur at a continuous transition rate, depending on the parameter δ, which expresses the percentage of multiparous and nulliparous cattle within the herd. For calibrating the progression rate δ, we considered the average percentage of nulliparous and multiparous cattle in a UK’s cattle herd, which according to [28] is 44% for cattle younger than 2 years old and 56% for the remaining ones. In order for our model to match these values, the progression rate was directly set to δ=1.28b. This value corresponds to heifers staying at the nulliparous state for about 470 days before the event of first parturition, that is in close agreement with [30] where the age before first parturition was estimated at about 410 days.

### Appendix A.4. Environmental Contamination Related Parameters

The contamination of the environment occurs from the infectious cattle $I_{mp}$ shedding *C. burnetii* through various pathways [23,24,25], except from milk in the UK. As discussed in Section 2.1.3, the infection is introduced to all stages $L_{j}^{i/o}$, except from the fully contaminated one. The contamination is uniformly distributed along every stage of higher contamination $L_{k}^{i/o}$ for k>j,j≠0 by a total indoor/outdoor shedding output. The shedding rate for the indoor environment is $p^{i}\left(t\right)\left(\eta_{i}I_{mp}+\eta_{b}bI_{mp}\right)L_{j}^{i}$, while for the outdoor is $p^{o}\left(t\right)\left(\eta_{o}I_{mp}\right)L_{j}^{o}$, since the term $\eta_{b}bI_{mp}$ related to shedding from birthing products does not contribute to outdoor landscape contamination. Similarly to the transmission rates, we adopted directly the parameter value of the indoor shedding rate $\eta_{i}=0.04\;$day^−1^ from [17] (who derived the parameter value from [7,12]), since they incorporated the same rate of inflow of the bacteria into the environment by shedding cattle (thus assuming a shedding rate). However, in [17] only one environmental landscape is considered with a shedding rate in the form $\eta_{i}I_{mp}$. In this work, since we consider multiple stages of landscape contamination, the same parameter value can be considered, this time with a shedding rate $\eta_{i}I_{mp}L_{j}^{i}$ (because $L_{j}^{i}$ are unitless and normalized to [0,1]). As for the indoor birth shedding rate, no direct parameter value was available in the literature. Thus, we calibrated $\eta_{b}$ so that the ratio of indoor shedding and birth shedding rate constants is $\eta_{i}/\eta_{b}=1/\left(2.7\times 10^{-6}\right)$ which is the ratio, found in [16], of the bacterium’s excretion in partus material per parturition over that in faeces and urine per day. Since in [16] this ratio is measured per parturition, we additionally multiplied $\eta_{b}$ with *b*; that is the inverse of the parturition interval. It is important here to note that in [16] the analysis was performed in Dutch dairy goat herds, thus this parameter is characterized by increased uncertainty. Nonetheless, its value compared to $\eta_{i}$ indicates a much higher excretion of the bacterium from partus rather than that from faeces. Similarly and for the same reasons, we adopted the framework of [17] to model the natural indoor environmental decay with the rate $K_{i}L_{j}^{i}$ and the active cleaning rate $\epsilon_{i}L_{j}^{i}$. We calibrated the parameter value of $K_{i}$ to match the one provided in [17] when considering only one landscape. Thus, we considered $K_{i}=0.0083/n\;$day^−1^, in the sense that when n=1, one retrieves the same parameter value. As for the active cleaning rate constant, we directly adopted the value $\epsilon_{i}=0.1\;$day^−1^ from [17]. Finally, since the outdoor environment operates similarly to the indoor environment and since no available data exist, we assumed that the outdoor shedding rate constant and the outdoor natural decay rate constant to have the same values with the indoors environment; i.e., $\eta_{o}=\eta_{i}$ and $K_{o}=K_{i}$, respectively.

## Appendix B. The Equilibria of the Q Fever Model

Here, we investigate the equilibria of the Q fever within-herd transmission model in Equations (1)–(5). Since the model involves the time-dependant parameters $p^{i}\left(t\right)$ and $p^{o}\left(t\right)$ in Equation (6), we account separately the case of only indoor housing (where $p^{i}\left(t\right)=1$ and $p^{o}\left(t\right)=0$) and that of only outdoors housing (where $p^{i}\left(t\right)=0$ and $p^{o}\left(t\right)=1$). As in Section 3, we consider n=5 stages of indoors/outdoors landscape contamination in Equation (7).

For deriving the disease-free equilibrium (DFE) of the model, we assume no initial infection in the herd; i.e., $I_{mp}^{*}=0$. The equations of the indoors and outdoors landscapes (Equations (4) and (5)) imply $L_{j}^{i*}=L_{j}^{o*}=0$ for j=1,…,n. From the conservation laws $\sum_{j=0}^{n}L_{j}^{i}=1$ and $\sum_{j=0}^{n}L_{j}^{o}=1$, it is easily derived that $L_{0}^{i*}=1$ and $L_{0}^{o*}=1$, respectively, indicating a contamination free indoors and outdoors environment at the DFE. Since $L_{j}^{i*}=L_{j}^{o*}=0$, it is implied that the overall transmission rate in Equation (1) is zero; i.e., $f(L_{j}^{i*},L_{j}^{o*})=0$. Thus, for determining the DFE, we solve the linear system of Equations (2) and (3) in the absence of infection:

$$\begin{array}{cccc} \rho E_{np}^{*}-\delta S_{np}^{*}+b\left(S_{mp}^{*}+E_{mp}^{*}\right)+b\left(1-p_{b}\right)A_{mp}^{*} & =0 & \rho E_{mp}^{*}+\delta S_{np}^{*}-\mu S_{mp}^{*} & =0\\ \left(\rho +\delta +\alpha \right)E_{np}^{*}+bp_{b}A_{mp}^{*} & =0 & \delta E_{np}^{*}-\left(\rho +\alpha +\mu \right)E_{mp}^{*} & =0\\ \alpha E_{np}^{*}-\delta A_{np}^{*} & =0 & \alpha E_{mp}^{*}+\delta A_{np}^{*}-\left(\tau +\mu \right)A_{mp}^{*} & =0\\ \; & \; & \tau A_{mp}^{*} & =0 \end{array}$$

Utilisation of the conservation law $S_{np}+E_{np}+A_{np}+S_{mp}+E_{mp}+A_{mp}+I_{mp}=1$ implies that the DFE is

(A1) $$\left(S_{np}^{*},E_{np}^{*},A_{np}^{*},S_{mp}^{*},E_{mp}^{*},A_{mp}^{*},I_{mp}^{*}\right)=\left(S_{np}^{*},0,0,S_{np}^{*}\delta /b,0,0,0\right)=\left(0.44,0,0,0.56,0,0,0\right)$$

accompanied by $\left(L_{0}^{i*},L_{1}^{i*},L_{2}^{i*},L_{3}^{i*},L_{4}^{i*}\right)=\left(1,0,0,0,0\right)$ for the indoors housing case and $\left(L_{0}^{o*},L_{1}^{o*},L_{2}^{o*},L_{3}^{o*},L_{4}^{o*}\right)=\left(1,0,0,0,0\right)$ for the outdoors housing case. As expected, the DFE reflects the data from DEFRA according to which the model was calibrated; the percentage of nulliparous cattle (younger than 2 years old) is about 44%, while that of multiparous cattle is about 56% in disease-free herds [28].

The endemic equilibrium of the model can be derived by solving the non-linear system of Equations (2)–(5) and the conservation laws, this time assuming $I_{mp}^{*}\neq 0$. Such a derivation cannot be fully derived by analytical means, thus we report the numerical solution of the equilibrium and only use analytic expressions for some variables of the model. In the indoors housing case, the equations of the indoors landscapes, Equation (4) and the conservation law $\sum_{j=0}^{n}L_{j}^{i}=1$ imply the following expressions of the equilibrium:

$$L_{4}^{i*}=f_{4}\left(I_{mp}^{*}\right),\;L_{3}^{i*}=f_{3}\left(I_{mp}^{*}\right)L_{4}^{i*},\;L_{2}^{i*}=f_{2}\left(I_{mp}^{*}\right)L_{3}^{i*},\;L_{1}^{i*}=f_{1}\left(I_{mp}^{*}\right)L_{2}^{i*},\;L_{0}^{i*}=1-L_{1}^{i*}-L_{2}^{i*}-L_{3}^{i*}-L_{4}^{i*}$$

where

$$f_{1}\left(I_{mp}^{*}\right)=\frac{I_{mp}^{*}}{X+Y+I_{mp}^{*}},\;f_{2}\left(I_{mp}^{*}\right)=\frac{X+2Y}{X+Y+I_{mp}^{*}},\;f_{3}\left(I_{mp}^{*}\right)=\frac{X\left(X+I_{mp}^{*}\right)+3\left(X+Y\right)Y}{\left(X+2Y\right)\left(X+Y+3I_{mp}^{*}\right)},$$

$$f_{4}\left(I_{mp}^{*}\right)=\frac{X\left(X+I_{mp}^{*}\right)\left(X+4Y+I_{mp}^{*}\right)+XYI_{mp}^{*}+Y^{2}\left(6X+4Y\right)}{\left(X+Y+I_{mp}^{*}\right)\left(X\left(X+I_{mp}^{*}\right)+3\left(X+Y\right)Y\right)},\;\mathrm{with}\;X=\frac{\epsilon_{i}}{\eta_{i}+\eta_{b}b}\;\mathrm{and}\;Y=\frac{K_{i}}{\eta_{i}+\eta_{b}b}$$

The overall transmission rate in Equation (1) obtains a rather complicated expression, thus making the derivation of $S_{np}^{*}$, $E_{np}^{*}$, $S_{mp}^{*}$ and $E_{mp}^{*}$ inaccessible. For the rest sub-populations, one can easily derive through Equations (2) and (3) the following expressions:

(A2) $$A_{np}^{*}=\frac{\alpha }{\delta }E_{np}^{*}\;I_{mp}^{*}=\frac{\tau }{\sigma +c+\mu }A_{mp}^{*}\;A_{mp}^{*}=\frac{\alpha (\sigma +c+\mu )}{c(\tau +\mu )+\mu (\sigma +\tau +\mu )}\left(E_{np}^{*}+E_{mp}^{*}\right)$$

Substitution of the parameter set, as listed in Table 1 with $p^{i}\left(t\right)=1$ and $p^{o}\left(t\right)=0$, results in the following expressions of the endemic equilibrium regarding the indoors landscapes

$$L_{4}^{i*}=\frac{I_{mp}^{*}}{4.11\cdot 10^{-3}+I_{mp}^{*}},\;L_{3}^{i*}=\frac{2.08\cdot 10^{-3}}{2.05\cdot 10^{-3}+I_{mp}^{*}}L_{4}^{i*},\;L_{2}^{i*}=\frac{1.37\cdot 10^{-3}+0.32I_{mp}^{*}}{1.37\cdot 10^{-3}+I_{mp}^{*}}L_{3}^{i*}$$

(A3) $$L_{1}^{i*}=\frac{4.36\cdot 10^{-6}+\left(3.12\cdot 10^{-3}+0.5I_{mp}^{*}\right)I_{mp}^{*}}{4.36\cdot 10^{-6}+\left(5.28\cdot 10^{-3}+I_{mp}^{*}\right)I_{mp}^{*}}L_{2}^{i*},\;L_{0}^{i*}=1-L_{4}^{i*}-L_{3}^{i*}-L_{2}^{i*}-L_{1}^{i*}$$

all of which are functions of $I_{mp}^{*}$. Note that assuming $I_{mp}^{*}=0$ one retrieves the DFE. The expressions in Equation (A2) for the endemic equilibrium now take the form:

(A4) $$A_{np}^{*}=22.32E_{np}^{*}\;A_{mp}^{*}=5.15\left(E_{np}^{*}+E_{mp}^{*}\right)\;I_{mp}^{*}=2.89A_{mp}^{*}$$

The expressions for $S_{np}^{*}$, $E_{np}^{*}$, $S_{mp}^{*}$ and $E_{mp}^{*}$ are very long to report here, thus we only report the numerical solution of Equations (2)–(5) for the endemic equilibrium in the indoors housing case

(A5) $$\begin{array}{cc} \left(S_{np}^{*},E_{np}^{*},A_{np}^{*},S_{mp}^{*},E_{mp}^{*},A_{mp}^{*},I_{mp}^{*}\right) & =(0.269,0.007,0.162,0.076,0.016,0.121,0.349)\\ \left(L_{0}^{i*},L_{1}^{i*},L_{2}^{i*},L_{3}^{i*},L_{4}^{i*}\right) & =(2.89\cdot 10^{-3},9.59\cdot 10^{-4},1.91\cdot 10^{-3},5.88\cdot 10^{-3},0.988) \end{array}$$

We next consider the case where the cattle herd is always housed outdoors (where $p^{i}\left(t\right)=0$ and $p^{o}\left(t\right)=1$). Despite being unfeasible, the endemic equilibrium of this case provides insight on the behaviour of the system in mixed housing scenarios, such as the seasonal housing case considered in the main text. For the outdoors housing case, the equations of the outdoors landscapes, Equation (5) and the conservation law $\sum_{j=0}^{n}L_{j}^{o}=1$ imply the following expressions of the endemic equilibrium:

$$L_{4}^{o*}=g_{4}\left(I_{mp}^{*}\right),\;L_{3}^{o*}=g_{3}\left(I_{mp}^{*}\right)L_{4}^{i*},\;L_{2}^{o*}=g_{2}\left(I_{mp}^{*}\right)L_{3}^{i*},\;L_{1}^{o*}=g_{1}\left(I_{mp}^{*}\right)L_{2}^{i*},\;L_{0}^{o*}=1-L_{1}^{o*}-L_{2}^{o*}-L_{3}^{o*}-L_{4}^{o*}$$

where

$$g_{1}\left(I_{mp}^{*}\right)=\frac{I_{mp}^{*}}{Z+I_{mp}^{*}},\;g_{2}\left(I_{mp}^{*}\right)=\frac{2Z}{Z+I_{mp}^{*}},\;g_{3}\left(I_{mp}^{*}\right)=\frac{3Z^{2}}{2Z(Z+3I_{mp}^{*})},\;g_{4}\left(I_{mp}^{*}\right)=\frac{6Z^{3}}{3Z^{2}\left(Z+I_{mp}^{*}\right)}$$

with $Z=K_{o}/\eta_{o}$. The expressions of the sub-populations in Equation (A2) are valid for the endemic equilibrium in the outdoors housing case, as well.

Substitution of the parameter set, as listed in Table 1 with $p^{i}\left(t\right)=0$ and $p^{o}\left(t\right)=1$, results in the following expressions of the endemic equilibrium regarding the outdoors landscapes

$$L_{4}^{o*}=\frac{I_{mp}^{*}}{4.15\cdot 10^{-2}+I_{mp}^{*}},\;L_{3}^{o*}=\frac{8.3\cdot 10^{-2}}{4.15\cdot 10^{-2}+I_{mp}^{*}}L_{4}^{o*},\;L_{2}^{o*}=\frac{2.08\cdot 10^{-2}}{1.38\cdot 10^{-2}+I_{mp}^{*}}L_{3}^{o*}$$

(A6) $$L_{1}^{o*}=\frac{8.3\cdot 10^{-2}}{4.15\cdot 10^{-2}+I_{mp}^{*}}L_{2}^{o*},\;L_{0}^{o*}=1-L_{4}^{o*}-L_{3}^{o*}-L_{2}^{o*}-L_{1}^{o*}$$

all of which are only functions of $I_{mp}^{*}$. Note that assuming $I_{mp}^{*}=0$ one again retrieves the DFE. The complete numerical solution of Equations (2)–(5) for the endemic equilibrium in the outdoors housing case is

(A7) $$\begin{array}{cc} \left(S_{np}^{*},E_{np}^{*},A_{np}^{*},S_{mp}^{*},E_{mp}^{*},A_{mp}^{*},I_{mp}^{*}\right) & =(0.436,1.28\cdot 10^{-4},2.87\cdot 10^{-3},0.542,8.17\cdot 10^{-4},4.86\cdot 10^{-3},0.014)\\ \left(L_{0}^{o*},L_{1}^{o*},L_{2}^{o*},L_{3}^{o*},L_{4}^{o*}\right) & =(9.36\cdot 10^{-2},0.127,0.224,0.302,0.253) \end{array}$$

## Appendix C. Confidence Level of the Model’s Response

The results presented in Section 3.1 were derived on the basis of the parameter set enlisted in Table 1. In order to increase the confidence level of the model’s response, we calculated the confidence intervals of the solution profiles of the model in the continuous housing case. Since the model in Equations (1)–(5) is deterministic, we generated multiple solutions with different parameter values. In particular, we sampled each parameter’s value in Table 1 in the interval [0.5p,1.5p] using a uniform distribution, where *p* is its nominal mean value. Since the values of $r_{i}$ and $r_{o}$ cannot surpass 1, we sampled them in the interval [0.6,1.0]. In addition, in order for the conservation laws to hold, we kept b=μ=1.28δ. Using this sampling approach, we generated 1000 parameter sets for the integration of the model in Equations (1)–(5).

Figure A1 depicts the prevalence of seronegative/seropositive cattle and the sub-population profiles for the continuous housing case, as in the left panel of Figure 3 and Figure 4, respectively. The solid curves indicate the mean value of the multiple solutions obtained with the 1000 different parameter sets, and the dashed curves indicate the 95% confidence intervals. It is clearly shown that 95% of the multiple solutions lie very close to the mean solution profile, indicating a high confidence level of the model’s response. In particular, the maximum deviation from the mean solution is reported at the 5th year, when (i) the prevalences of seronegative, seropositive, seropositive non-shedding and seropositive shedding cattle are 37.8 ± 0.6%, 62.2 ± 0.6%, 28.6 ± 0.45% and 33.6 ± 0.3%, respectively, and (ii) the sub-populations of $S_{np}$, $E_{np}$, $A_{np}$, $S_{mp}$, $E_{mp}$, $A_{mp}$, $I_{mp}$ are 26.9 ± 0.35%, 0.728 ± 0.017%, 16.25 ± 0.35%, 8.5 ± 0.25, 1.7 ± 0.04%, 12.35 ± 0.24% and 33.6 ± 0.3%, respectively.
